# Fabrication and biological evaluation of 3D-printed calcium phosphate ceramic scaffolds with distinct macroporous geometries through digital light processing technology

**DOI:** 10.1093/rb/rbac005

**Published:** 2022-02-22

**Authors:** Jing Wang, Yitao Tang, Quanle Cao, Yonghao Wu, Yitian Wang, Bo Yuan, Xiangfeng Li, Yong Zhou, Xuening Chen, Xiangdong Zhu, Chongqi Tu, Xingdong Zhang

**Affiliations:** National Engineering Research Center for Biomaterials, College of Biomedical Engineering, Sichuan University, Chengdu 610064, China; National Engineering Research Center for Biomaterials, College of Biomedical Engineering, Sichuan University, Chengdu 610064, China; National Engineering Research Center for Biomaterials, College of Biomedical Engineering, Sichuan University, Chengdu 610064, China; National Engineering Research Center for Biomaterials, College of Biomedical Engineering, Sichuan University, Chengdu 610064, China; Department of Orthopaedics, West China Hospital of Sichuan University, Chengdu 610041, China; National Engineering Research Center for Biomaterials, College of Biomedical Engineering, Sichuan University, Chengdu 610064, China; National Engineering Research Center for Biomaterials, College of Biomedical Engineering, Sichuan University, Chengdu 610064, China; Department of Orthopaedics, West China Hospital of Sichuan University, Chengdu 610041, China; National Engineering Research Center for Biomaterials, College of Biomedical Engineering, Sichuan University, Chengdu 610064, China; National Engineering Research Center for Biomaterials, College of Biomedical Engineering, Sichuan University, Chengdu 610064, China; Department of Orthopaedics, West China Hospital of Sichuan University, Chengdu 610041, China; National Engineering Research Center for Biomaterials, College of Biomedical Engineering, Sichuan University, Chengdu 610064, China

**Keywords:** digital light processing (DLP), 3D printing, pore structure, osteoinduction

## Abstract

Digital light processing (DLP)-based 3D printing technique holds promise in fabricating scaffolds with high precision. Here raw calcium phosphate (CaP) powders were modified by 5.5% monoalcohol ethoxylate phosphate (MAEP) to ensure high solid loading and low viscosity. The rheological tests found that photocurable slurries composed of 50 wt% modified CaP powders and 2 wt% toners were suitable for DLP printing. Based on geometric models designed by computer-aided design (CAD) system, three printed CaP ceramics with distinct macroporous structures were prepared, including simple cube, octet-truss and inverse face-centered cube (fcc), which presented the similar phase composition and microstructure, but the different macropore geometries. Inverse fcc group showed the highest porosity and compressive strength. The *in vitro* and *in vivo* biological evaluations were performed to compare the bioactivity of three printed CaP ceramics, and the traditional foamed ceramic was used as control. It suggested that all CaP ceramics exhibited good biocompatibility, as evidence by an even bone-like apatite layer formation on the surface, and the good cell proliferation and spreading. A mouse intramuscular implantation model found that all of CaP ceramics could induce ectopic bone formation, and foam group had the strongest osteoinduction, followed by inverse fcc, while cube and octet-truss had the weakest one. It indicated that macropore geometry was of great importance to affect the osteoinductivity of scaffolds, and spherical, concave macropores facilitated osteogenesis. These findings provide a strategy to design and fabricate high-performance orthopedic grafts with proper pore geometry and desired biological performance via DLP-based 3D printing technique.

## Graphical Abstract

**Figure:**
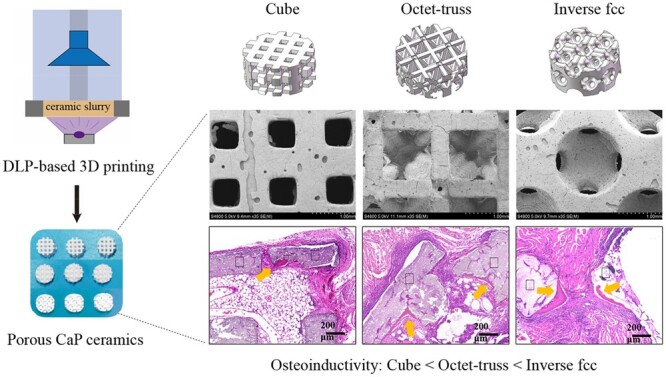

## Introduction

Due to the similarity of chemical composition to the inorganic component of nature bone, calcium phosphate (CaP) ceramic has been widely applied as bone substitute material to repair and regenerate bone tissue. Previous literature has demonstrated that CaP ceramics with proper chemical composition and geometric structure exhibit not only biocompatibility and osteoconductivity, but also osteoinductivity, namely, the ability to induce ectopic bone formation in non-osseous site. Moreover, three-dimensional (3D) macroporous structure of CaP ceramic scaffold is generally considered as one of the prerequisite parameters for its osteoinduction. Thus far, a variety of approaches have been used to fabricate porous bioceramics, including freeze casting, slurry foaming, foam impregnation and so on [1, 2]. However, these traditional methods are difficult to accurately control the pore size, shape and distribution of porous scaffolds.

Recently, 3D printing technology holds great promise in simultaneously constructing external configuration and internal porous structure of scaffolds, according to the model of computer-aided design (CAD) [3–5]. Different types of 3D printing technology, such as stereolithography (SL), fused deposition modeling (FDM), selective laser sintering (SLS), show great differences in printing accuracy. Among them, SL has great advantages to achieve good precision, which utilizes liquid photopolymer resin cured by light to solidify the pattern layer by layer to create 3D scaffolds. Moreover, digital light processing (DLP) can be adopted in SL system, using high-resolution digital light projector as a light source [6], to make fluid slurry layer-by-layer polymerization under the irradiation of ultraviolet light. This principle of surface exposure curing breaks through the traditional technology to realize the formation of curved surface, porous structure and complex geometry, which is conducive to rapid fabrication of scaffolds with fine structure and small deformation [7]. A majority of studies focused on using DLP system to construct industrial ceramics (e.g. alumina, silicon), while, there were only sporadic reports on preparation of CaP bioceramics via DLP [7, 8]. For instance, our previous study used DLP-based SL technology to fabricate CaP bioceramics with controllable 3D structure and high accuracy through layer-by-layer photopolymerization of photocurable slurry, which was comprised of CaP powders and photosensitive resin [9].

After 3D printing, the CaP ceramic green bodies are needed to be sintered at high temperature to eliminate additives such as resins and other additives. Thus, a high solid loading in the printing slurry is required to achieve high-performance ceramic scaffolds, which exhibit good precision, intact structure and proper mechanical features, by avoiding the excessive shrinkage during sintering process. However, the slurry viscosity rises rapidly with the increase of powder solid content, which is detrimental to the formation of ultra-thin printing layer through the slurry spreading. In order to endow the slurry with both high powder solid content and good rheological property, surface modification of ceramic powders has been used to increase the dispersibility of hydrophilic ceramic powders in hydrophobic photosensitive resin [10]. Our previous study compared the effects of four surfactants, including stearic acid, oleic acid, sebacic acid and monoalcohol ethoxylate phosphate (MAEP) on the surface modification of CaP powders, suggesting that MAEP was the best one, as it resulted in the lowest slurry viscosity [9]. Therefore, in present study, MAEP was chosen as modifier to prepare the photocurable slurry.

The traditional porous CaP ceramic used in the orthopedic area is often fabricated by the H_2_O_2_ foaming method, which suffers from unsatisfactory mechanical strength and poor tailorability, thereby only filling and repairing bone defects in non-loading bearing sites. In addition, it is difficult to accurately control pore structure and size distribution of the foamed CaP ceramic, which limits the further optimization of its biological properties. The emergence of 3D printing technology promotes the design and fabrication of bone substitute, as it can achieve personalized customization and precise control of 3D structure, especially for irregular and complex structure to match the bone defects [11]. However, compared with the foamed ones, the 3D-printed CaP bioceramics often exhibited the relatively weaker bone-repairing ability. For instance, Barba *et al.* [12] implanted the 3D-printed and foamed hydroxyapatite (HA) ceramics into the femur of beagle dogs, showing significantly fewer new bones observed in the 3D-printed ceramics than the foamed ones. It suggested that the divergence of their osteogenic abilities might be attributed to the differences in their macropore architecture and surface microstructure. Extensive studies have demonstrated that the foamed porous ceramics, presenting a large number of interconnected spherical pores in the diameter of a few hundred micrometres, often exhibit excellent osteoconductivity and osteoinductivity [13–15]. However, there is almost no report in the literature to fully recapitulate macropore geometry of the foamed CaP ceramics via 3D printing. In addition, microporous structure also plays a critical role in the bioactivity of CaP ceramics, especially in their osteoinductivity [13, 14]. Thus, to endow the bioceramics with abundant micropores, the edible toner served as a pore-forming agent to be induced into the printing slurry, due to its safety, non-toxicity and good fluidity.

**Fig. 1.**
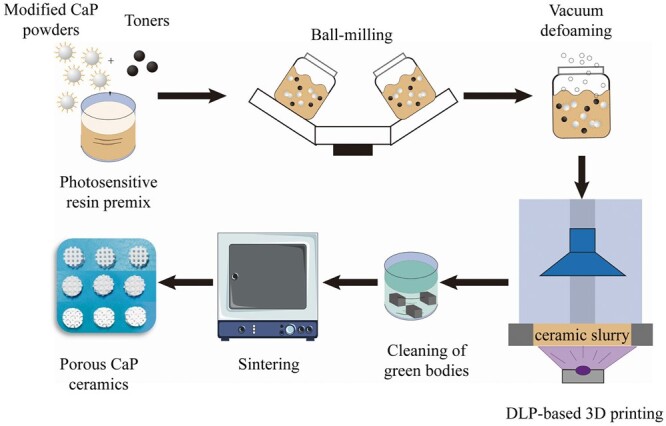
Schematic diagram of DLP-based 3D printing for the preparation of porous CaP ceramics

In this study, to obtain 3D-printed CaP bioceramics with high performance, surface modification of CaP powders was optimized to prepare the proper photocurable slurry with high powder solid loading and low viscosity, and then DLP-based 3D printing technique was performed to fabricate ceramic green bodies based on three self-designed geometric models, including cube, octet-truss and inverse face-centered cube (fcc) following the fabrication procedures shown in Fig. 1. After debinding and sintering, the final CaP bioceramics were obtained and characterized. Further, the *in vitro* cell experiment and *in vivo* animal study were carried out to compare the bioactivity, especially the osteoinductivity of three 3D-printed porous CaP ceramics with different macroporous structure. The foamed CaP ceramic with good osteoinductive capacity was used as the control. The experimental data confirmed the close correlations between macropore geometry and biological performance, which might provide guidance for the design of bone substitute materials in the future.

## Materials and methods

### Modification of calcium phosphate powders

The raw CaP powders were provided by Sichuan Baiameng Bioactive Materials LLC (Chengdu, China). The wet modification method was adopted to modify raw CaP powders by using MAEP as the surfactant. The different amount of MAEP was dissolved into absolute ethanol by ultrasonically stirring for 15 min to prepare MAEP solution with different concentrations (0.5%, 1.0%, 1.5%, 2.0%, 2.5%, 3.0%, 3.5%, 4.0%, 4.5%, 5.0%, 5.5%, 6.0% and 6.5%). Then, CaP raw powders and MAEP solutions (1:1) were ground and mixed thoroughly by ball milling at the speed of 300 rpm for 8 h. The CaP suspensions were dried in an oven at 60°C for 24 h, ground into fine powders, and sifted through a 200-mesh sieve to finally get the surface-modified CaP powders. Then, the particle sizes and distribution of CaP powders before and after modification were examined by laser particle analyzer (ZS90, Malvern, US) and specific surface area analyzer (Gemini VII 2390t, Micromeritics, USA). The raw and modified CaP powders (0.3 g) were pressed into dense discs by using uniaxial pressing (FY-15, Tianhe, China), and their surface wettabilities were measured by using an optical contact angle apparatus (Kono’s SL200KS, USA).

### Preparation of modified CaP ceramic slurries

In order to increase the number of micropores in 3D-printed ceramic, toner powders (Wanglin Biological, China) were used as pore-forming agent in this study. The different amounts of surface-modified CaP powders and toners were mixed with the photosensitive resin, which was prepared by adding the photoinitiator into the monomer with a ratio of 1–49. Then the mixtures were ball milled thoroughly by using a planetary ball mill (PBM-V-4L, DECO, China) at 300 rpm for 4 h. The bubbles in the mixed slurries were removed by using a vacuum defoaming mixer (MV-300, Marath, China) at 1200 rpm for 3 min. A variety of photocurable slurries with the different mass fractions of CaP powders (40, 45, 50, 55, and 60 wt%) and toners (0.5%, 1.0%, 1.5%, 2.0%, 2.5% and 3.0%) were prepared, and their viscosities were measured by using a viscometer (DV2T, Brookfield, USA) to determine the optimal solid loading of CaP powders in slurries.

### Photopolymerization properties of CaP ceramic slurries

The photopolymerization properties of CaP slurries are mainly determined by critical exposure intensity (*E*_c_) and projected depth (*D*_p_). According to the Beer-Lambert Law, the curing thickness of single layer of slurry in the DLP system was calculated based on the theoretical equation as follows:

$$C_{d}=D_{p}\left(\ln\;E-\ln\;E_{c}\right)$$

where *C*_d_ is the curing depth of slurry monolayer (μm), and *E* is the exposure intensity of UV light incident on the surface of slurry (mJ/cm^2^).

The curve of ln *E* (X-axis) versus *C*_d_ (Y-axis) was plotted and linearly fitted to get the photocuring parameters (*D*_p_ and E_c_) of CaP slurries using Origin Pro 8.5 software.

### Fabrication of CaP ceramics

In this study, the photocurable CaP slurries with high solid content, low viscosity and low toxicity were subjected to the 3D printing by using an Autocera-M photocurable DLP printer (TEN DIMENSIONS, China) with the initial wavelength of 405 nm. In brief, three geometric models were constructed by a CAD system (3D CAD Design Software, Dassault Systemes SolidWorks Corp, USA), including simple cube, octet-truss, and inverse fcc. The designed inverse fcc used inverted densely packed fcc structure as template, with spherical pores at the eight corners and the center of six surfaces. The designed model structure file was saved in STL format, imported into model processing software (10 Dim, TEN DIMENSIONS, China) suitable for Autocera-M printer, and then sliced to ultrathin layers with a thickness of 50 μm. The 3D printing was carried out to prepare the ceramic green bodies. Through moving a scraper blade back and forth, a ultrathin layer of photocurable slurry was evenly formed, and selectively exposed to UV light at the wavelength of 405 nm for curing. This procedure was repeated until the formation of the intact green bodies. After the printing, the CaP ceramic green bodies were immersed in 100% ethanol for 15 min ultrasound to thoroughly clean the unsolidified slurry, and dried at room temperature. Subsequently, the ceramic green bodies were heated using muffle furnace to remove the additives such as photosensitive resins, defoaming agents and carbon powders, but avoid the generation of cracks and other defects. The thermogravimetric analysis (TGA) was performed on the green bodies with heating from room temperature to 600°C at a heating rate of 5°C/min under air atmosphere by using TGA/DSC2 analyzer (Mettler toledo, Switzerland). Based on the TGA curve, an optimized sintering procedure was established to obtain the resultant CaP ceramics with different pore structures.

### Characterization of 3D-printed CaP ceramics

The macroscopic morphology of green bodies and CaP ceramics was observed by using a stereomicroscope (SteREO Discovery V20, ZEISS, Germany). The geometric changes of ceramics before and after sintering were measured to evaluate the printing precision and heat shrinkage. The microscopic morphology of CaP ceramics were visualized by using a field emission scanning electron microscope (FE-SEM, JSE-5900LV, Japan). The phase compositions of raw powders, modified powders and CaP ceramic were analyzed by using X-ray diffraction (XRD, Shimadzu XRD-6100, Japan), and the obtained peaks were compared with the standard references for HA (09-0432) and β-TCP (09-0169). The porosity, pore number and pore distribution were tested by using a mercury intrusion porosimetry (MIP, AutoPore IV 9500, Micromeritics). The compressive strength of CaP ceramics (10 × 10 × 10 mm^3^, *n* = 3) was tested by using a universal testing machine (Precision Universal Tester Autograph AGX, Japan) at the loading speed of 1 mm/min.

### In vitro experiments

To evaluate their bioactivity, the 3D-printed CaP ceramic discs with the model design dimension (Φ9 × 3 mm^3^), and the dimension after sintering (Φ6.3 × 2.1 mm^3^). For comparison, the H_2_O_2_-foamed CaP ceramic discs with the similar dimension and phase composition were used as the control. These foamed CaP ceramics were provided by Sichuan Baiameng Bioactive Materials LLC (Chengdu, China) based on the similar procedure mentioned in previous studies [16]. The four groups of CaP ceramics were named as cube, octet-truss, inverse fcc and foam, respectively. Then CaP ceramic discs were subjected to dry heat sterilization 250°C for 2 h before experiment.

#### Bone-like apatite formation

Simulated body fluid (SBF) immersion was performed to evaluate the bone-like apatite forming ability of the 3D-printed CaP ceramics. After being immersed into SBF at 37°C for 3 days, the CaP ceramics were taken out, gently washed with deionized water, and dried overnight at 60°C. Then, SEM was used to observe the bone-like apatite formation on the CaP ceramic surface.

#### Cell culture and seeding

Murine bone marrow mesenchymal stem cells (BMSCs), which were purchased from Cyagen Biosciences Inc. (Suzhou, China), were cultured in α-MEM medium (Gibco, USA) with 10% fetal bovine serum (FBS, Gibco, USA) and 1% penicillin/streptomycin (Gibco, USA) at 37°C in a humidified atmosphere of 5% CO_2_. After 90% confluence, BMSCs were trypsinized and seeded onto CaP ceramic discs with a cell density of 2 × 10^4^ cells/disc for cell proliferation and morphology, and 1 × 10^5^ cells/disc for gene expression. The culture media were changed twice per week.

#### Cell proliferation and morphology

The cell proliferation (*n* = 3) was measured using the AlamarBlue assay (Invitrogen, USA) at days 1, 3 and 7. Following the manufacturer’s instructions, after removal of the culture medium, the samples were washed with PBS and incubated with fresh medium with 10% AlamarBlue reagent for 2 h at 37°C, and then 100 μl aliquots of the supernatants were transferred to a 96-well plate. The optical density was analyzed at a wavelength of 570 and 600 nm using a multifunctional, full-wavelength microplate reader (Varioskan Flash, Thermo Scientific, USA).

After culturing for 1, 3 and 5 days, BMSCs grown on CaP ceramics were stained with fluorescein diacetate and propidium iodide (PI, Topbio Science, China), and visualized under a confocal laser scanning microscopy (CLSM, TCS SP5, Leica, Germany). Meanwhile, to observe cell spreading, the samples were stained with Alexa Fluor 488 conjugated-phalloidin (1:200, Yuheng Biotech, Suzhou, China) for cytoskeletal protein and DAPI (1:1000, Sigma) for cell nuclei, and examined under a CLSM. BMSCs grown on CaP ceramics were also observed by using SEM.

#### Gene expression

Osteogenesis-related gene expression of BMSCs grown on CaP ceramics (*n* = 3) was measured using a real-time quantitative reverse transcription PCR reaction (qRT-PCR). In brief, at days 1, 7 and 14, the total RNA (*n* = 3) was extracted using a Rneasy Mini Kit (Qiagen, Germany) and transcribed into complementary DNA (cDNA) by using an iScript cDNA Synthesis Kit (Bio-Rad, USA). Then, q-PCR was performed to measure the mRNA levels of several osteogenesis-related genes by using a CFX96t real-time PCR detection system (CFX960, Bio-Rad, USA) with SoFastt EvaGreens Supremix (Bio-Rad, USA), including runt related transcription factor 2 (Runx-2), BMP-2 (bone morphogenetic protein-2), alkaline phosphatase (ALP), bone sialoprotein (BSP), osteopontin (OPN), and osteocalcin (OCN). The gene primers are listed in Supplementary Table S1. The ΔΔCt method was used to calculate the relative expression of each target gene by normalizing its cycle threshold value to that of the housekeeping gene—GAPDH using a Bio-Rad CFX Manager 3.0 system.

### Animal study

**Fig. 2.**
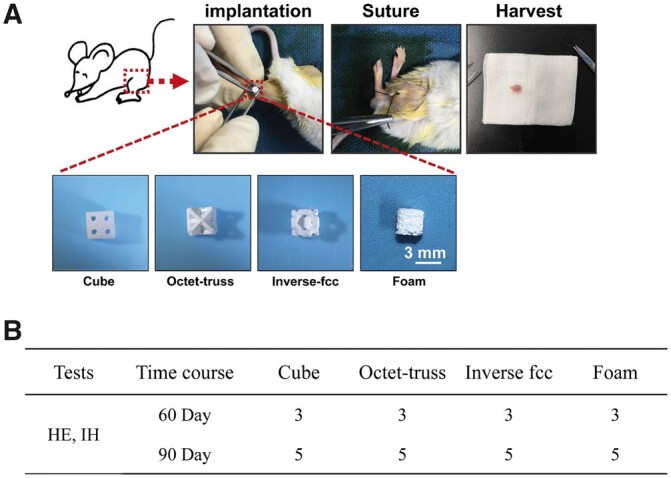
(**A**) The surgical procedures for intramuscular implantation of CaP ceramics in BALB/C mice. (**B**) The number of implants per CaP ceramic at each time point

The osteoinductivity of 3D-printed CaP ceramics was evaluated using a murine intramuscular implantation model. All animal study was approved by the local Animal Care and Use Committee of Sichuan University and followed the guidelines for the Protection and use of Experimental Animals published by the National Academy of Science, China. As shown in Fig. 2A, each 4-week-old BALB/C mouse was anesthetized by pentobarbital sodium, and bilateral muscle pouches were created by blunt dissection of the rectus femoris. Then the 3D-printed CaP ceramic cube with a dimension of 2.8 × 2.8 × 2.8 mm^3^ was surgically inserted into each pouch. At 60 and 90 days post-surgery, five specimens of each CaP group were harvested, fixed in 4% paraformaldehyde, decalcified with 10% EDTA (pH 7.4), dehydrated, and embedded in paraffin. The specimens were sectioned with 5 μm in thickness. Three sample sections of each specimen were stained with hematoxylin/eosin (HE) and toluidine blue (TB) for histological observation. After mounting, the stained slides were imaged using a Panoramic 250/MIDI scanner (3D HISTECH, Hungary) with a Caseviewer 2.0 software. Images were evaluated histomorphometrically using Image-Pro Plus software (IPP, Media Cybernetic, USA). The area of newly formed bone in the available pore space, namely the proportion of bone area was quantitatively measured based on 15 randomly selected sections from each ceramic group. Meanwhile, immunohistochemical (IHC) staining for osteogenic proteins (i.e. BMP-2 and OCN) was also carried out to assess the osteogenic ability of CaP ceramics with different structures.

### Statistical analysis

All quantitative results were obtained from at least three parallel measurements for each group, and the obtained data were expressed as mean ± standard deviation (SD). Statistical analysis was performed using one-way analysis of variance (ANOVA) in SPSS 16.0 software (SPSS Inc., USA). The significance level of *P* < 0.05 was set as significant difference.

## Results

### Characterization of modified CaP powders

**Fig. 3.**
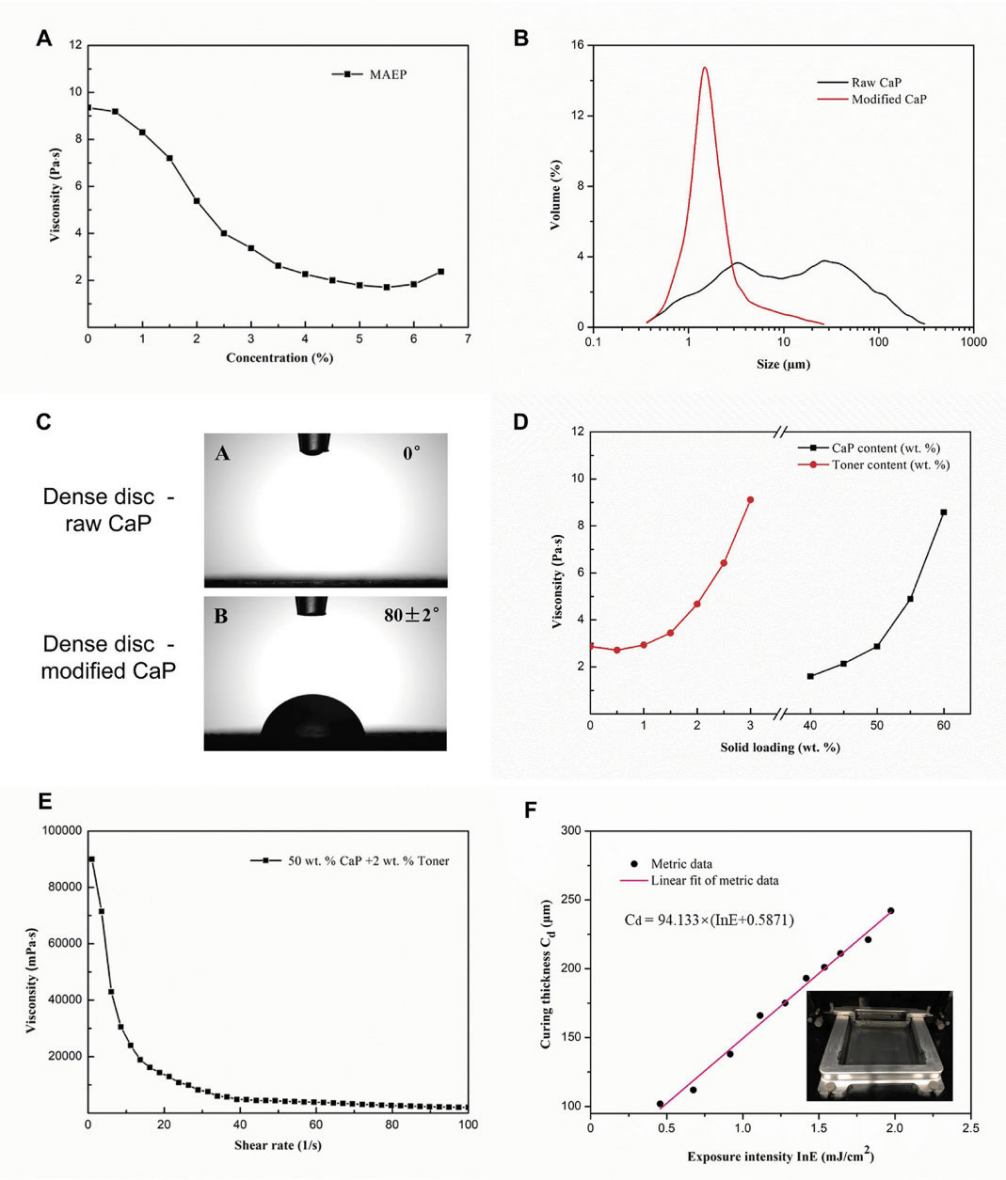
(**A**) Rheological test for the 40 wt% CaP solid loading slurries varying with the surfactant (MAEP) concentration at 30 s^−1^ shear rates. (**B**) Particle size distribution of CaP powders before and after modification (raw CaP powders and modified CaP powders). (**C**) Contact angles of dense CaP discs made of raw CaP powders and modified CaP powders. (**D**) Viscosities of modified CaP slurries varying with the solid loadings of different CaP contents and toner contents at 30 s^−1^ shear rates. (**E**) Viscosity change of the slurries containing 50 wt% CaP and 2 wt% toner (C) at different shear rates. (**F**) The curve of curing thickness (*C*_d_) of the slurry versus exposure intensity (ln *E*). (Insert) the spreading state of solid loading slurry in the printer cartridge after a movement of double blade in bin

Based on our previous study [9], MAEP was chosen as a surfactant to modify the raw CaP powders, which was then used as the main component of photocurable slurry. The rheological tests evaluated the effect of MAEP concentrations on the viscosity of ceramic slurry. It was found that within a certain MAEP content range (<5.5%), the slurry viscosity of the 40 wt% powder solid loading slurry decreased with the increase of MAEP content at 30 s^−^^1^ shear rates, and the rate of decline varies from slow to fast, reaching a plateau at about 5% MAEP content (Fig. 3A). Therefore, MAEP with a concentration of 5.5% was selected as the modifier parameter in this study.

After modified by 5.5% MAEP and ball milled for 8 h, the particle size and distribution of CaP powders had a distinct change. The results (Fig. 3B and Table 1) demonstrated that particle size distribution of raw CaP powders ranged widely from 0.3 to 300 μm, and the average particle size was 13.30 μm, which was difficult to meet the requirement of photocuring DLP. Whereas, the particle size of modified CaP powders decreased significantly to a range of 0.4–10 μm with an average size as 2.37 μm, which was suitable for the printing process. In addition, the specific surface area of CaP powder was 16.70 m^2^/g before modification (raw CaP) and 28.38 m^2^/g after modification (modified CaP). The MAEP modification also significantly increased the values of contact angles from 0° to 80 ± 2° (Fig. 3C and Table 1), indicating that the powders transmitted from hydrophilic to hydrophobic state, which could greatly improve the solid loading in the subsequent photocurable slurry.

**Table 1.** The particle size, specific surface area and contact angle of CaP powders before and after modification

	Average particle size (μm)	Specific surface area (m^2^/g)	Contact angle (°)
Raw CaP	13.32	16.6989	0
Modified CaP	2.37	28.3816	80 ± 2

### Characterization of photocurable slurry

As shown in Fig. 3D, the viscosities of CaP slurries varied with the increase of modified CaP and toner powder contents. When the solid loading of CaP powders ranged from 40 to 50 wt%, a relatively stable rise of the slurry viscosities was observed. Whereas, once the CaP contents exceeded 50 wt%, the slurry viscosities raised sharply. Moreover, the viscosity curve of toner contents showed that the slurry viscosities decreased slightly from 0 to 0.5 wt%, and then increased greatly, especially when the toner contents exceeded 2%. It is known that high slurry viscosity is unfavorable for the spreading of slurry and the formation of ultrathin layer. To make the photocurable slurry with high solid loading and low viscosity, 50 wt% CaP and 2 wt% toner were selected in this study, and its rheological property was measured. As shown in Fig. 3E, following the increase of shear rate, the slurry viscosity had a sharp decrease at the initial stage (<10 s^−^^1^), then declined slowly, and finally maintained a relatively stable state (> 50 s^−^^1^). The results suggested that this slurry presented a typical shear thinning feature, which is believed to favor the slurry spreading during DLP. In addition, the photopolymerization property of slurry with 60 wt% CaP was measured to obtain the curve of Cd versus exposure intensity (ln *E*) in Fig. 3F. According to the formula in the figure, the critical exposure intensity (*E*_c_) was 0.5560 mJ/cm^2^ and the corresponding depth of projection (*D*_p_) was 94.133 μm. The thickness of the single-layer slice in the model should be less than the *D*_p_ value, and the exposure intensity in printing device should be greater than the *E*_c_ value. Thus, in this study, the slice thickness of the 3D printing ceramic model was set as 50 μm, which met the DLP-based printing requirements.

### Characterization of 3D-printed ceramics

TGA analysis was performed on the 3D-printed ceramic green bodies. The TG curves (Fig. 3B) had a slight decrease between 100 and 250°C, which was due to the evaporation of residual crystal water in the ceramic green body. The mass loss mainly occurred at 250°C–550°C, as the total mass loss was around 50%, suggesting that the decomposition of photosensitive resins and toners happened within this range. There was no further mass loss above 550°C, indicating that the modifier, resin monomer, initiator and toner were completely removed by sintering, and only CaP powders were left in the final ceramics. Based on these findings, the debinding/sintering process was set to decompose all additives and maintain the geometric structure of CaP ceramics, as shown in Fig. 3D. At the initial stage, a low heating rate of 1°C/min was adopted from room temperature (RT) to 250°C, and the crystalline water was completely eliminated by holding at 250°C for 2 h. During the stage of 250°C–550°C, the green body was heated at a rate of 1°C/min and kept at 550°C for 2 h to ensure that the resins and toners could be completely burned out. Then it was heated to 1100°C at a rate of 3°C/min and held for 2 h. Finally, the samples were collected after the furnace cooled to RT.

**Fig. 4.**
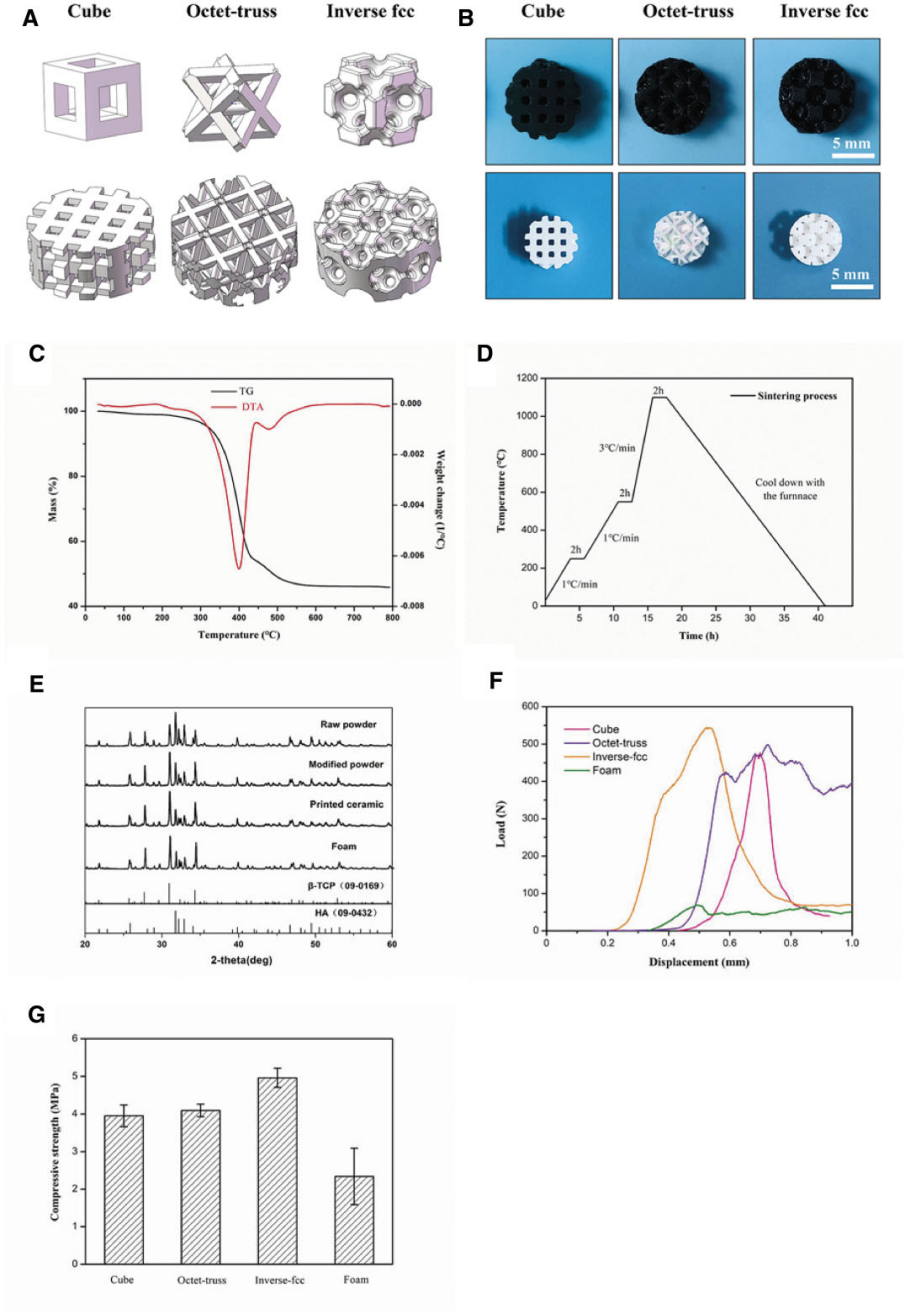
(**A**) The primitive units and 3D models designed by CAD system. (**B**) Stereoscopic microscopy images of 3D-printed CaP ceramics before and after sintering. (**C**) Thermal analysis curve of the ceramic green body. (**D**) Debinding and sintering process of the ceramic green bodies. (**E**) XRD patterns of raw CaP powders, modified CaP powders, and 3D-printed CaP ceramics. (**F**) Compression stress-displacement curves and (**G**) average compressive strengths of four CaP ceramics with different structures

The images of stereoscopic microscopy showed the macroscopic appearances of 3D-printed green and sintered ceramic bodies (Fig. 4B). It was found that the geometric structure of both ceramics was intact with no obvious defects and cracks. In both X–Y and Z directions, the green bodies completed matched the designed model (Fig. 4A and B). The dimensions of the designed models, green bodies and sintered ceramics are listed in Table 2, suggesting that our DLP system presented a high 3D printing precision with an accuracy ≥99%, and a large shrinkage of ceramic bodies was also observed after sintering. Moreover, the shrinkage rates of sintered ceramics compared to green bodies of cube, octet-truss and inverse fcc were comparable (*P > *0.05), with the values of about 30% in either X–Y or Z direction, presenting an isotropic shrinkage. The porosity of the designed models and the porosity of the sintered ceramic blocks based on the theoretical calculation was also listed in Table 2, showing that the porosity of the sintered blocks was close to that of the designed models, which also verified the high precision of DLP system in this study. XRD patterns of raw CaP powders, modified CaP powders, and 3D-printed CaP ceramics were shown in Fig. 4E, and the obtained diffraction peaks were identified by the standard references for HA (JCPDS: 09-0432) and β-TCP (JCPDS: 09-0169). It was found that printed CaP ceramics were only composed of β-TCP and HA without other components, indicating that the modifiers, resins and toners were completely removed without residue left after sintering. The phase composition ratios of HA to β-TCP were 0.62:0.38 in raw powders, 0.40:0.60 in modified powders, 0.33:0.67 in printed CaP ceramics and 0.35:0.65 in formed CaP ceramics, respectively. The mechanical properties of three printed CaP ceramics were also measured. Figure 4F and G showed the compression stress–displacement curves and average compressive strengths of four CaP ceramics, with the values of 3.95 ± 0.29 MPa for cube, 4.09 ± 0.17 MPa for octet-trus, 4.96 ± 0.26 MPa for inverse fcc and 2.34 ± 0.75 MPa for foam, respectively.

**Table 2.** Dimensions, shrinkage rates and porosity of designed models, green bodies and sintered CaP ceramics with different structures

Sample		Cube	Octet-truss	Inverse fcc	Foam
Model geometric dimension (mm)	X/Y-axis	9	9	9	—
	Z-axis	3	3	3	—
Green body (mm)	X/Y-axis	9.03 ± 0.06	8.97 ± 0.05	9.00 ± 0.05	—
	Z-axis	3.01 ± 0.03	2.99 ± 0.02	3.01 ± 0.02	—
Ceramic (mm)	X/Y-axis	6.28 ± 0.09	6.33 ± 0.11	6.31 ± 0.10	—
	Z-axis	2.11 ± 0.07	2.04 ± 0.04	2.10 ± 0.05	—
Shrinkage rate (%)	X/Y-axis	30.51 ± 1.36	29.46 ± 0.97	29.97 ± 1.43	—
	Z-axis	30.10 ± 1.87	31.77 ± 1.57	28.01 ± 2.42	—
Porosity (%)	Models	70	72	74	—
	Reality	68.36 ± 1.89	70.27 ± 1.74	73.45 ± 1.67	74.83 ± 2.59

**Fig. 5.**
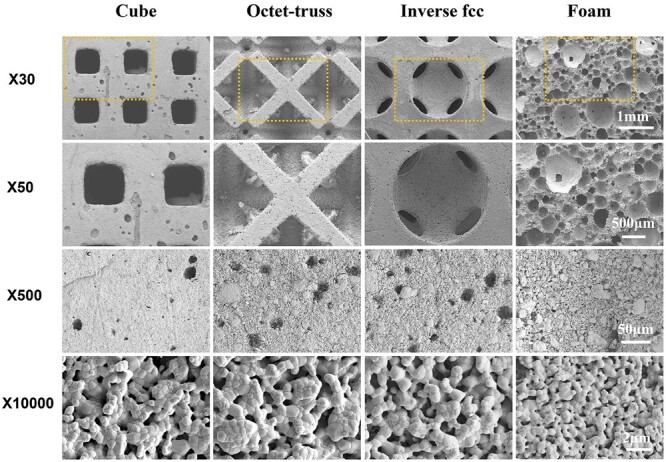
SEM images of porous CaP ceramics made by 3D printing (cube, octet-truss, inverse fcc) and H_2_O_2_ foaming (foam)

The typical SEM images showed the microstructure of printed ceramics with different model structures in Fig. 5. All ceramics had porous structure, presenting large and interconnected pores (>500 μm), no obvious defects and cracks, as well as no clogged and closed pores. A large number of pits were visible in the pore walls of printed ceramics, which were caused by the pore-forming agents (i.e. toner powders) that were burned out during sintering. In high magnification images, the fusion of grains with distinct grain boundaries was observed, with the grain sizes ranged from 0.5–1.2 μm, and abundant nano- to sub-micron-sized pores existed. The formed CaP ceramics (foam) had abundant macropores (50–500 μm) with grain sizes of 0.2–1.0 μm.

**Fig. 6.**
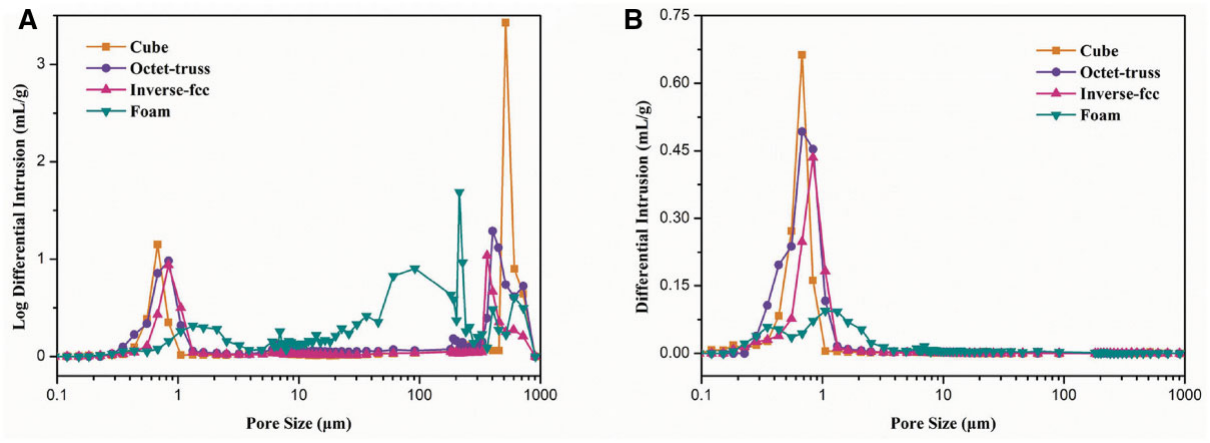
(**A**) Pore size distributions and (**B**) pore quantity distributions of 3D-printed CaP ceramics

MIP was carried out to analyze the pore structure of 3D-printed CaP ceramics with different structures. Figure 6A presents the pore size distribution to characterize the macroscopic and microscopic pore structure. It was found that cube ceramics had macro-pores of about 700 μm with narrow distribution, reflecting the designed regular square-shaped pores, while, octet-truss and inverse fcc had the similar size distribution of macro-pores in a range of 300–720 μm, as the irregular structure designed in both models could form macropores of different dimensions. The three 3D-printed CaP ceramics also exhibited the similar distribution of minor-pores (∼10 μm) and micro-pores (0.3–2 μm), which was consistent with SEM images. The formed CaP ceramics (foam) showed a wider pore distribution with macro-pores in a range of 20–800 μm and micro-pores of sizes in 0.5–3 μm. Figure 6B showed the pore quantity distributions, finding that micro-pores (<1 μm) had significant advantages in the 3D-printed CaP ceramics, while, micro-pores (<3 μm) were predominant in the formed CaP ceramics.

### Ability of bone-like apatite formation

**Fig. 7.**
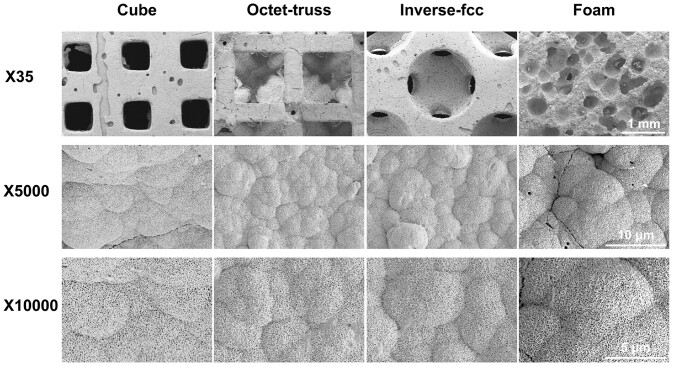
SEM images of bone-like apatite formed on CaP ceramics after immersion in SBF for 3 days

To evaluate their *in vitro* bioactivity, the 3D-printed CaP ceramics were immersed into SBF for 3 days in 37°C, and the formation of bone-like apatite was observed by SEM. As shown in Fig. 7, SBF immersion could not alter the macroscopic morphology of all CaP ceramics generated by 3D printing and H_2_O_2_ foaming, but promote the deposition of bone-like apatite onto their surface. It was observed that the surface of CaP ceramics was completely covered by a layer of reticulated crystals.

### Cell proliferation and spreading

**Fig. 8.**
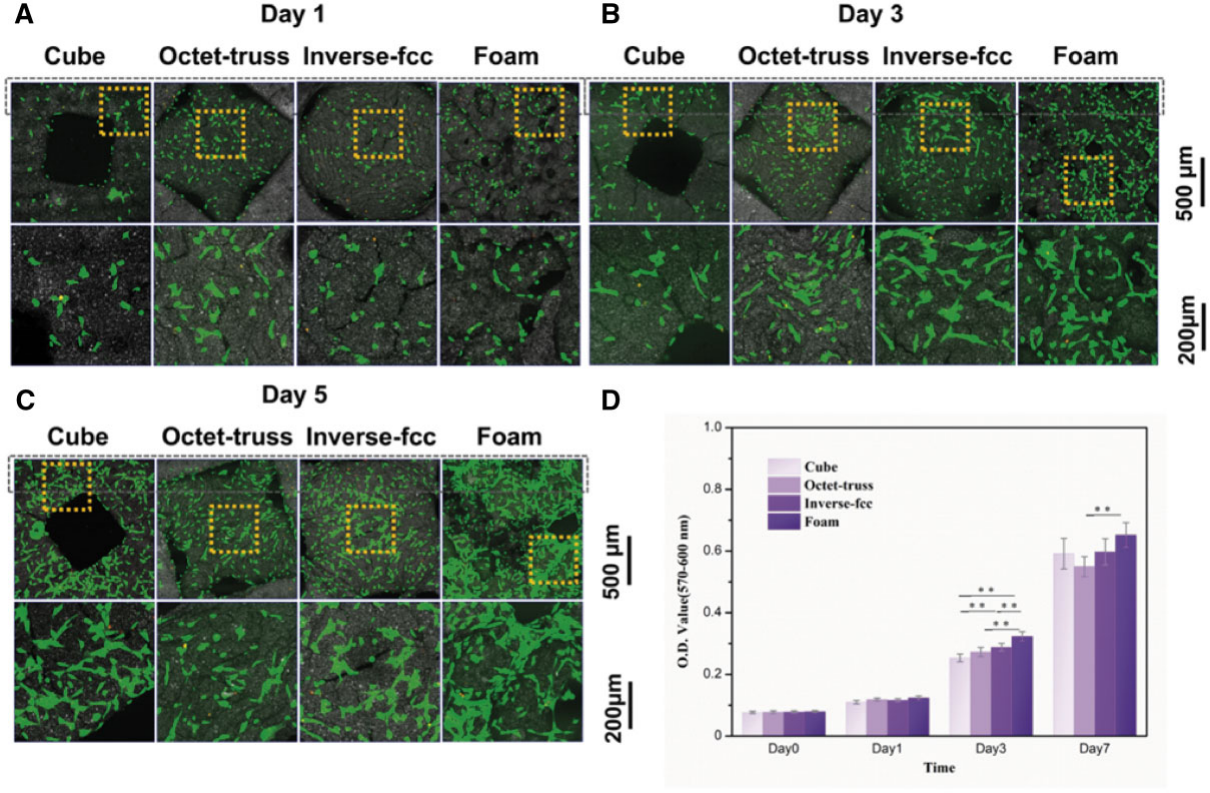
CLSM observations for BMSCs growth onto CaP ceramics at (**A**) day 1, (**B**) day 1 and (**C**) day 5. (**D**) AlamarBlue results for the proliferation of BMSCs on CaP ceramics from day 0 to day 7. ** refers to *P* < 0.01

As shown in Fig. 8, the CLSM observations found that BMSCs could adhere and spread well along the pore walls of CaP ceramics, presenting normal cell morphology (green), while a few dead cells (red) were observed. Moreover, the number of BMSCs gradually increased from day 1 to day 5, and BMSCs gradually connected to each other to form extensive cell-cell interactions. The significantly more cells were seen on H_2_O_2_-foamed CaP ceramics (i.e. foam) than the three 3D-printed ones (i.e. cube, octet-truss and inverse fcc), which was consistent with the AlamarBlue results. It suggested that all of CaP ceramics exhibited good biocompatibility to support the proliferation and spreading of BMSCs.

### Cell morphology

**Fig. 9.**
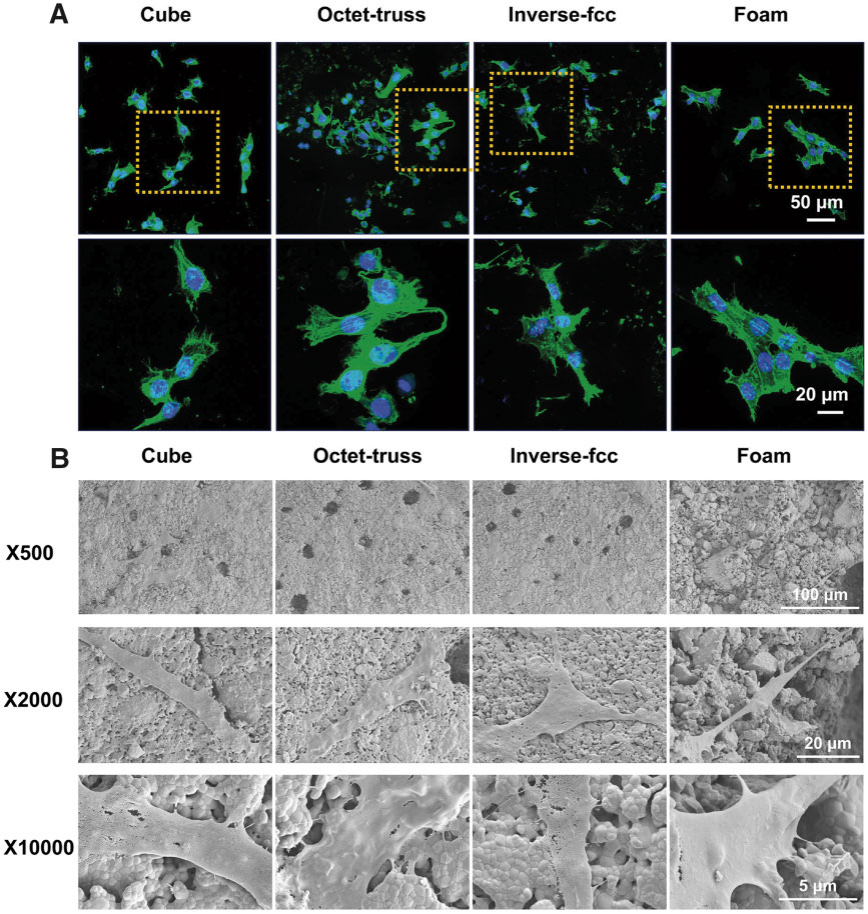
(**A**) Representative CLSM images of phalloidin staining and (**B**) SEM images for BMSCs cultured in CaP ceramics. Green fluorescence: filament-actin; blue fluorescence: DAPI-stained cell nuclei

CLSM observation of F-actin phalloidin staining (Fig. 9A) found that BMSCs grown on CaP ceramics with different structures showed the similar cell morphology, presenting the spindle and polygonal shapes. SEM images (Fig. 9B) further confirmed that BMSCs adhered closely to the surface of CaP ceramics and spread well after culturing for 2 days. Many cells presented a typical spindle-like shape and outstretched spread pseudopods with a plenty of filopodia on the cell edges to firmly grasp the crystal grains of CaP ceramics.

### Expression of osteogenic genes

**Fig. 10.**
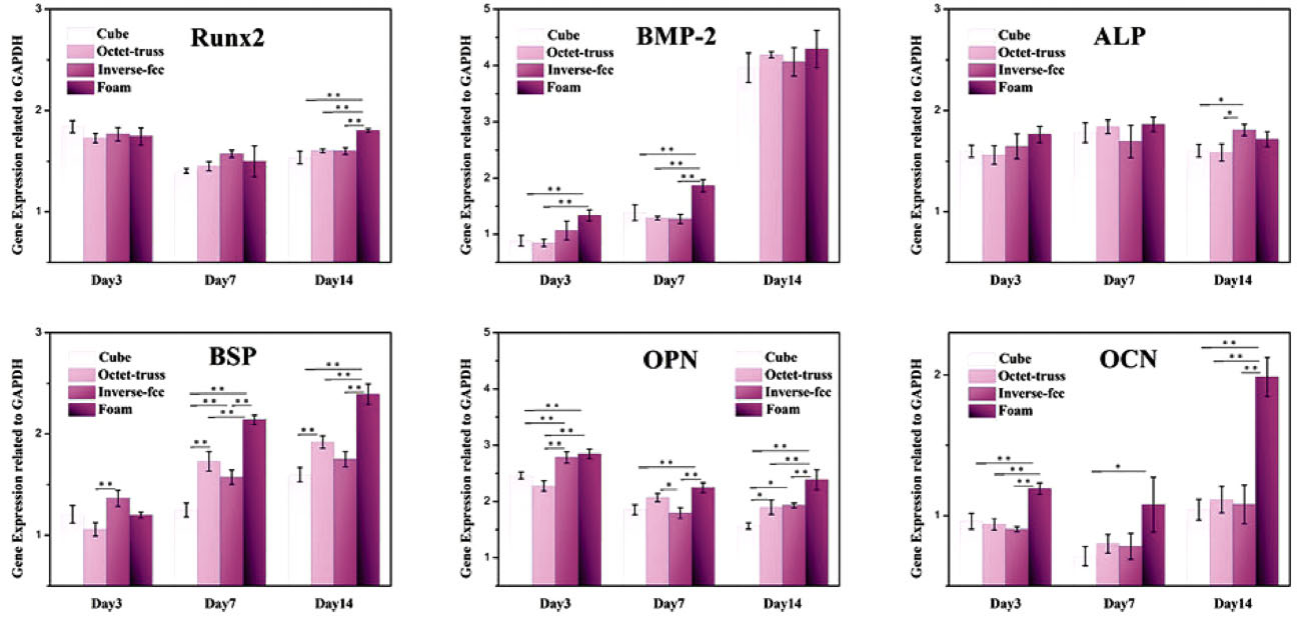
The expression of osteogenic genes (Runx-2, BMP-2, ALP, BSP, OPN and OCN) in BMSCs cultured on CaP ceramics for 3, 7 and 14 days. * refers to *P* < 0.05, and ** refers to *P* < 0.01

The qRT-PCR analysis was used to quantitatively investigate the expression of osteogenic genes (Runx2, BMP-2, ALP, BSP, OPN and OCN) by BMSCs grown on the four CaP ceramics with different structures after 3, 7 and 14 days of co-culture *in vitro*. As shown in Fig. 10, the expression of BMP-2, BSP and OCN genes presented the similar trend, as their expression levels increased with prolonged culture time, while, the expression levels of Runx2, ALP and OPN genes did not change significantly at each time point. The highest gene expression was seen in foam group (H_2_O_2_-foamed CaP ceramics) for BMP-2 gene at days 3 and 7, BSP gene at days 7 and 14, and OPN and OCN genes during the whole culture period. Moreover, compared to cube, octet-truss ones, inverse fcc 3D-printed group showed the significantly higher expression of ALP gene at day 14, BSP gene at day 3 and OPN gene at days 3 and 14.

### In vivo osteoinductivity

**Fig. 11.**
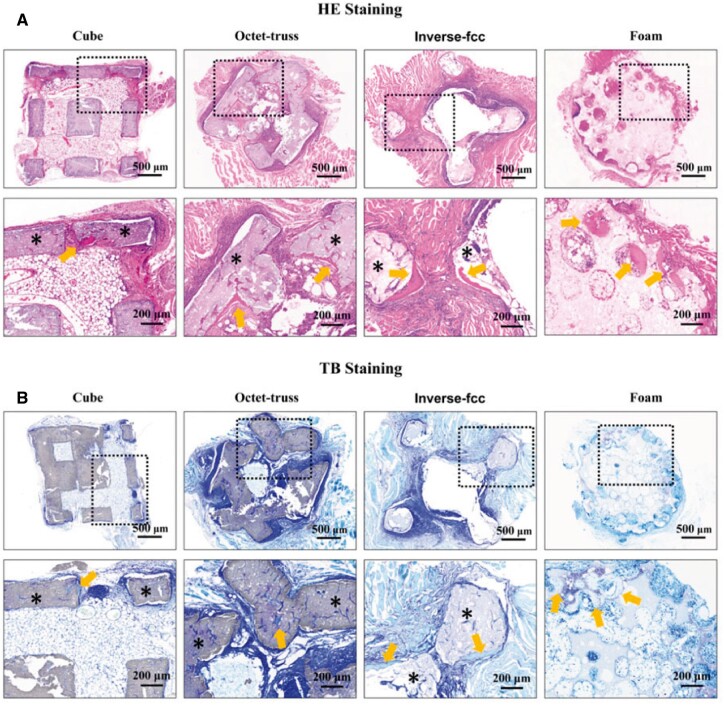
(**A**) HE and (**B**) TB staining for CaP ceramics after intramuscular implantation for 90 days. ∗: CaP ceramics; arrows: newly formed bones

Osteoinductivity of four CaP ceramics was compared based on a murine intramuscular implantation model. The histological analyses of the retrieved samples were performed after 60- and 90-day implantation, and the occurrence probability of ectopic bone formation induced by each CaP ceramic was listed in Table 1. The histomorphometrical results demonstrated that at 60 days post-surgery, only one of three samples (1/3) in H_2_O_2_-foamed group (foam) had new bone formation, while no osteogenesis (0/3) occurred in all of 3D-printed groups. At 90 days, ectopic bone formation was observed in one of five (1/5) in cube and octet-truss groups, two of five (2/5) in inverse fcc group and four of five (4/5) in foam group. HE staining (Fig. 11) showed that sporadic new bone tissues were observed along the pore walls of CaP ceramics, as evidenced by dense osteoid tissues with abundant collagen-rich matrix and bone lacunae resided by mature osteocytes.

There was only one sign of osteogenesis at the defect on the ceramic surface of cube group. Two distinct new bone regions were observed in octet-truss and inverse fcc groups with different locations, as in octet-truss group, bone formation occurred in the corner and the concave surface of pores, while, in inverse fcc group, new bones surrounded the convex surface of cylindrical ceramic skeletons. In foam group, the newly formed bones grew along the wall of spherical ceramic pores. The proportion of newly formed bone in the available pore space (bone area/pore area) was listed in Table 3, with the values from high to low as Foam = Inverse fcc > Octet-truss > Cube.

**Table 3.** The occurrence probability of ectopic bone formation induced by each CaP ceramic and the proportion of bone area in the osteoinductive specimens

Materials		Cube	Octet-truss	Inverse fcc	Foam
Bone induction	60 days	0/3	0/3	0/3	1/3
	90 days	1/5	1/5	2/5	4/5
The proportion of bone area (%)	60 days	0	0	0	0.27
	90 days	0.11	0.73	0.97 ± 0.33	1.07 ± 0.20

Images of TB staining at 90 days after implantation showed that there was no obvious fibrous encapsulation around four ceramic groups. New tissues filled almost all of the pores and even grew into the interspaces of ceramic skeletons, which might be created by the pore-forming agent or the degradation of ceramics. Bone tissues stained in light blue were observed, which was basically consistent with the findings of HE staining.

**Fig. 12.**
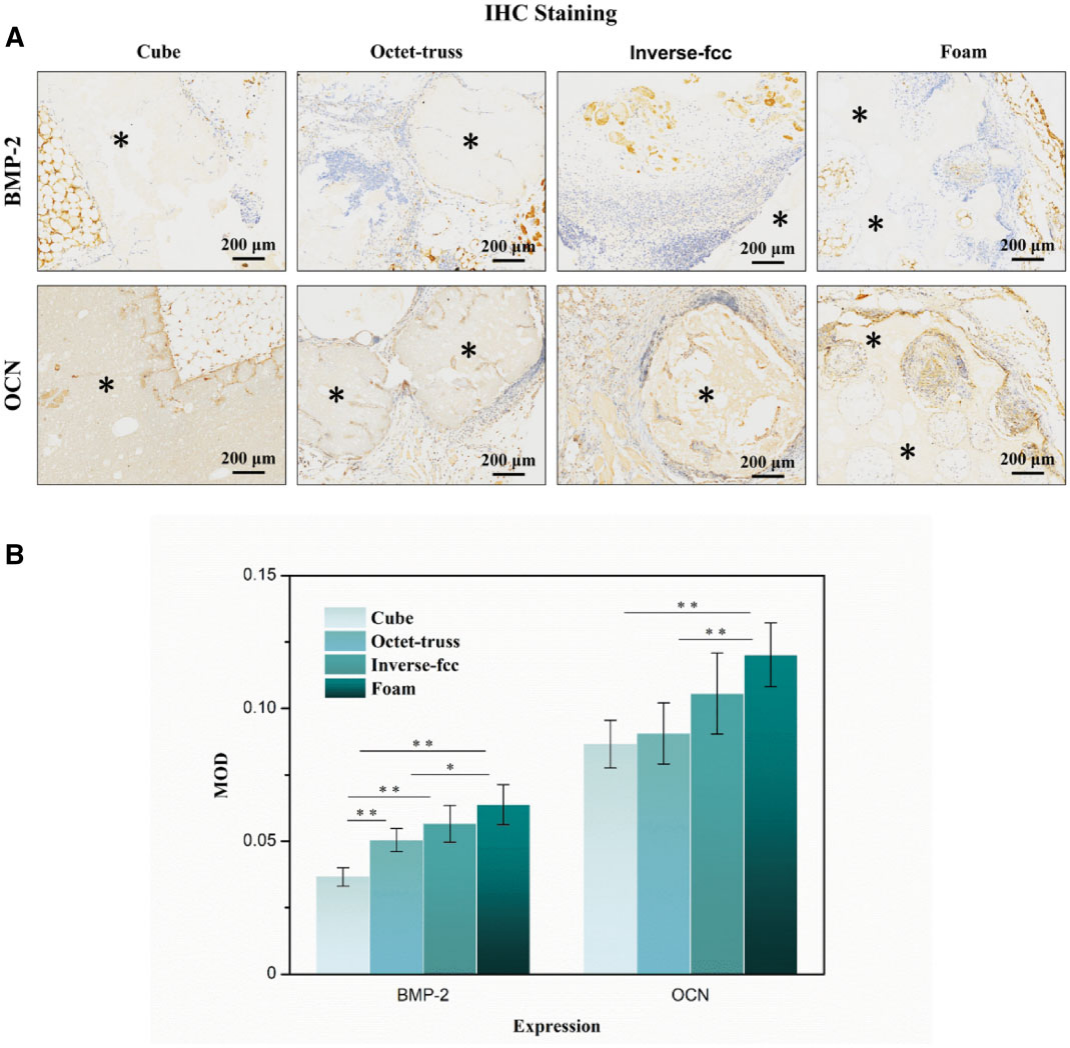
(**A**) IHC staining of BMP-2 and OCN in the decalcified sections of CaP ceramics after intramuscular implantation for 90 days. The brown color: positive expression. (**B**) Quantitative analysis for BMP-2 and OCN present in four specimens. * refers to *P* < 0.05 and ** refers to *P* < 0.01

IHC staining was used to investigate the expression of specific osteogenic proteins (i.e. BMP-2 and OCN) in the specimens at day 90 after implantation. As shown in Fig. 12A, the positive expressions of BMP-2 and OCN were identified in the tissues grown into the inner macropores of four ceramics. Further quantitative analysis of the IHC-stained sections (Fig. 12B) suggested that compared to cube and octet-truss ones, foam and inverse fcc specimens exhibited significantly higher expression levels of BMP-2 and OCN proteins (*P* < 0.05).

## Discussion

DLP-based 3D printing holds great promise for constructing porous CaP bioceramics with high performance. The photocurable slurry, composed of CaP ceramic powders and photosensitive resin, plays a critical role in ensuring successful printing. Generally speaking, CaP powders have good hydrophilicity, while the photosensitive resin monomers and photoinitiators often show hydrophobic feature. The hydrophobic resin will inevitably hinder the dispersion of hydrophilic CaP powder and decrease the solid loading. Low powder solid loading in the printing slurry could result in the excessive shrinkage, poor printing accuracy and weak mechanical property of the 3D-printed scaffolds. Previous literature has demonstrated an effective approach to increase the powder solid loading in photocurable slurry through surface modification of CaP powders to convert their wettability from hydrophilic to hydrophobic [17–19]. Surfactants have been widely used to change the affinity between organic and inorganic phases, as they can reduce aqueous solution surface tension or liquid–liquid interfacial tension [20]. According to the findings of our previous research, MAEP was selected to modify CaP powders in this study. Our results showed that the viscosity of ceramic slurry decreased with the increase of MAEP content until it reached 5.5%, resulting from the significantly reduced surface hydrophilicity of CaP powders, which was verified by contact angle tests. Then, the viscosity of ceramic slurry raised slightly when the MAEP content was in a range of 5.5–6.5%, which might be because that the concentration of surfactant (MAEP) exceeded the saturation adsorption value of powders, and the entanglement or bridging of the excess surfactant molecules would cause agglomeration, leading to an increase in the slurry viscosity [20]. Thus, 5.5% MAEP was chosen as the optimal surfactant to achieve the lowest slurry viscosity for the further study.

In order to obtain the high-performance CaP ceramics with good precision and mechanical property, it is necessary to increase the solid content of CaP powder as much as possible. At the same time, the photocurable slurry should meet the requirements of DLP printing, suggesting that the slurry should also have good fluidity. In present study, the rheological analyses found that when the solid loading of CaP powders was below 50 wt%, the viscosity of slurry raised slowly, while when it was higher than 50 wt%, the slurry viscosity increased dramatically. Meanwhile, edible toners were added as pore-forming agent in the slurry to construct microporous structure. When the toner content was in a range of 0–0.5 wt%, the slurry viscosity decreased slightly, which might be due to the self-lubricity of the toner, which further improved the slurry fluidity. Then, the slurry viscosity maintained a slow-rising state from 0.5 to 2 wt%. A sharp increase of slurry viscosity was observed when the toner content was greater than 2 wt%, which was not appropriate for printing. These findings indicated that 50 wt% CaP powder and 2 wt% toner were the best choice for the preparation of the printing slurry, whose rheological properties were further investigated. The rheological curve showed that the printing slurry with 50 wt% solid loading and 2 wt% toner had the typical shear thinning characteristics. During the photocuring process in the DLP system, the printing slurry could be evenly spread in the tank with the double-channel scraper moving back and forth.

Other rapid prototyping methods (such as FDM and SLS) often use the extrusion additive manufacturing technology, which is prone to a large size error between the mold and printed samples, especially for the pore structure [21, 22]. By contrast, theoretically speaking, DLP system should have a greater printing accuracy, as it uses surface exposure molding, which primarily depends on the spreading and photopolymerization of printing slurry [7, 8, 20]. Reasonable curing parameters are beneficial to improving the printing accuracy of ceramic body [23]. Based on the curve of *C*_d_ versus ln *E*, the optimal parameters were set as 50 μm of slice thickness, 11.55 mW/cm^2^ of light intensity, and 2 s of exposure times in the DLP system.

The phase compositions of the original powder, modified powder and sintered ceramic were analyzed by XRD, showing that there were only two phases (i.e. HA and β-TCP), but their proportion HA:β-TCP ratios gradually decreased from 0.62:0.38 in raw powders, to 0.40:0.60 in modified powders and finally to 0.33:0.67 in printed CaP ceramics. It might be due to the phosphorus element in the aliphatic alcohol ether phosphate ester MAEP, which caused the conversion of HA phase to β-TCP phase [9].

Numerous studies have demonstrated that the porosity of 60–80%, interconnected macropores of spherical shape (100–800 μm), mesopores with tens of microns and abundant nano/micropores (<10 μm), are generally considered as the prerequisite factors for the osteoinductivity of CaP ceramics [23]. Therefore, to construct 3D-printed CaP ceramics with high performance, three porous CaP ceramics with different porous structures were prepared by DLP printing, which could basically meet the structural requirements of osteoinduction mentioned above, including simple square pores (cube), octet-truss and inverse fcc structures. The cube scaffold was chosen in this study, as its tetragonal structure in cubic pore shape has been considered as the most widely used structure in 3D printing [24]. The octet-truss scaffold has structural unit with the regular octahedral centroid surrounded by eight tetrahedrons and distributed on its corresponding eight faces, thereby exhibiting cubic symmetry and isotropic lattice structure to simulate the trabecular structures of natural bone. The inverse fcc scaffold exhibited the structural unit with the densely stacked inverted fcc lattice structure as a template, and spherical pores at the eight corners and the center of the six faces, which could mimic the spherical pore structure of foamed ceramic [25]. SEM and MIP analyses found that macroscopic pores of the 3D-printed CaP ceramics completely replicated those of the designed model, and there were also a number of micropores (∼10 μm) and nano-to-micron-sized pores (0.3–2 μm), which were caused by the decomposition of resins and carbon powders. The results suggested that the three 3D-printed scaffolds had the same phase composition and the similar surface microstructure, but exhibited the different macroporous structure. Literature has demonstrated that the porosity and pore structure of 3D-printed scaffolds play a critical role in affecting their mechanical properties [24], and the compressive strength of scaffold is often negatively correlated with the porosity [26, 27]. Our results found that in spite of its higher porosity, inverse fcc group showed a higher compressive strength than cube and octet-truss groups, which might be due to the influence of pore structure. Compared to the cubic pores in cube group and the cubic, triangular, irregular pores in octet-truss group, inverse fcc group exhibited a unique pore structure with spherical pore geometry, as previous studies suggested that the scaffolds with spherical macropores showed a superior mechanical properties than the ones with cubic macropores [28, 29]. Moreover, the 3D-printed CaP ceramics showed a significantly higher compressive strength than the foamed one. It might be because that H_2_O_2_ foaming caused the loose scaffold stents equipped with a large number of micropores in foam group, while, DLP-based 3D printing technique produced the scaffolds with the relatively denser stents.

Moreover, pore structure is believed as one of the critical material factors in determining the cell behaviors and affecting the tissue regeneration [24, 30, 31]. In this study, the bioactivity, particularly osteoinductivity of 3D-printed scaffolds with different pore structure was further evaluated *in vitro* and *in vivo*. Inducing new bone formation is a complex process involving the interaction of scaffold materials, local microenvironment, cells and tissues. When a bioactive material is immersed in SBF, Ca and P ions in the solution would deposition on the substrate surface to form a layer of bone-like apatite, which is similar to the inorganic component of natural bone tissue. At present, the formation of bone-like apatite on the surface has been widely used to evaluate if the biomaterial has bioactivity *in vitro* [32]. In this study, after soaking in SBF for 3 days, the surfaces of three 3D-printed and foamed CaP ceramics were fully covered with apatite layer, indicating that they all had good biological activity.

Moreover, BMSCs, which show pluripotency to differentiate into multiply cell lineages under appropriate cues [33], have been widely used to evaluate tissue inductivity of a biomaterial scaffold. Differentiation of BMSCs into osteoblasts involves the stages of adhesion, proliferation, extracellular matrix deposition and matrix mineralization [34]. In this study, phalloidin staining and SEM observation found that BMSCs were tightly attached to the ceramic surface and spread out well, with no obvious difference between three printed groups (cube, octet-truss and inverse fcc). This might be because that cell adhesion and spreading were closely related to the surface micro/nano- structure [35], which were comparable among three printed ceramics. CLSM observation and AlarmBlue assay showed that the number of BMSCs increased dramatically with the extension of culture time, and significantly more cells were found in foam group than three printed groups at day 7. It might be due to the occurred non-through-hole structure of foam ceramics, which offered more area for cell growth.

It is well-known that osteogenesis is regulated by a series of osteogenic genes and proteins. Runx2, also known as Core-binding factor alpha 1 (Cbfα1) is one of the key transcription factors that is essential for the differentiation of stem cells into osteoblasts [36, 37]. ALP is constitutively expressed by the osteoblast during the matrix maturation phase to hydrolyze inorganic pyrophosphate to generate phosphate for the deposition of bone mineral, and thus the expression level of ALP is one of the specific markers of early osteogenesis [38]. BMP-2 is an important bone growth factor that induces MSCs to differentiate into osteoblast precursors and stimulates their differentiation into mature osteoblasts, increasing ALP production and collagen matrix synthesis [39, 40]. As one of the most abundant noncollagenous proteins in bone, BSP is synthesized by osteoblasts with functions in bone mineralization processes and in regulation of osteoblast-mineral matrix binding [41, 42]. OPN is a constituent protein in the bone matrix, which interacts with different molecules to mediate matrix mineralization and bone formation [43, 44]. OCN is specifically synthesized and secreted by differentiated and mature osteoblasts, and is closely related to osteoblast activation [45, 46]. Therefore, these factors were used as specific osteogenic markers to evaluate the ability of CaP ceramics to induce the osteoblastic differentiation of BMSCs *in vitro*. The qRT-PCR results showed that foam group had the highest expression of several osteogenic genes (i.e. Runx2, BMP-2, BSP, OCN and OPN) in BMSCs. Among three printed ceramic groups, inverse fcc group partially up-regulated the expression of ALP, BSP and OPN genes, while cube group often down-regulated the expression of these osteogenic genes. It indicated that macropore structure of CaP ceramics could significantly affect the osteogenic differentiation of BMSCs.

Moreover, the *in vivo* evaluation was also performed using a mouse intramuscular implantation model [47, 48], showing that ectopic bone formation could be observed in all ceramics at day 90 post-implantation, which indicated that all of CaP ceramics had the ability of osteogenic induction. Histomorphometrical analysis and IHC staining further found that three printed and foamed CaP ceramics exhibited different osteoinductive capacities, which was consistent with our *in vitro* results, as foam group had the strongest osteoinduction, followed by inverse fcc, while cube and octet-truss had the weakest osteoinduction. Differences in osteoindction between them might be due to the distinct macropore structure and surface microtopography. Compared to three 3D-printed ones, foamed ceramics (foam) had some non-through-holes and smaller pore diameter, which might weaken the fluidity of body fluids in the ceramic scaffold, and thus favor the formation of local high ionic microenvironment and even the local protein accumulation, thus promoting the osteoblastic differentiation of BMSCs [49, 50]. Besides, foam group also had different surface topography with significantly smaller grain size, and previous studies demonstrated that osteoinduction of bioceramics was improved with a decrease in grain size [13, 47, 51].

The *in vivo* evaluation showed that among the three printed ceramics, inverse fcc group displayed the highest osteoinductive ability, which might be due to its similarity to spherical pore structure of foam one. It is believed that pore geometry, namely pore shape, pore size, pore surface area and local curvature, is of great importance to strongly influence cell adhesion, proliferation, migration and differentiation [52–55]. The tissue growth is also strongly affected by the geometrical features of pores or channels in 3D scaffolds, as cells are able to sense curvature with sizes much larger than those of the cells themselves. Rumpler *et al.* [52] found that regardless of the original shape, tissue growth formed the rounding of pore corners surrounding a spherical central opening, suggesting that tissue amplification on pore or channel faces was greater in hexagonal than square, followed by triangular one. Barba *et al.* used a canine intraosseous implantation model, showing that spherical, concave macropores of the foamed scaffolds contributed to an enhanced bone regeneration, compared to the traditional 3D-printed scaffolds with orthogonal-patterned struts and prismatic, convex macropores [12, 56]. Our study was in excellent agreement with these findings, as different from cube group with the square pores and octet-truss group with the triangle pores, inverse fcc group simulated the macroporous geometry of foamed porous ceramics (foam) with spherical, concave pores, leading to an accelerated bone formation in non-osseous site.

These findings demonstrated that although three printed CaP ceramics showed weaker osteoinduction than those of the foamed CaP ceramics. However, inverse fcc group exhibited higher *in vitro* and *in vivo* osteoinduction than other two ones, and almost had the close osteogenic ability with foam one. Therefore, further optimization on improving the osteoinductivity of 3D-printed CaP ceramics via various approaches could be quite hopeful. For instance, considering the important role of pore geometry in the bioactivity of scaffolds, the novel printing model may be designed to better simulate macroporous structure of the foamed ceramics, such as constructing an upgraded inverse fcc model with the reduced pore size. In addition, surface modification may be applied to increase surface micro/nano-topography or enhance surface bioactivity through incorporation of active factors.

## Conclusion

DLP-based 3D printing technique was performed to fabricate three high-performance CaP ceramics with high precision and good biological properties, using surface modified CaP powders containing 50 wt% solid loading, 5.5% MAEP, and 2 wt% toner, and the optimized photocuring parameters. These printed CaP ceramics exhibited the similar phase composition and surface microstructure, but the different macropore geometries. Based on *in vitro* cell experiment and *in vivo* murine intramuscular implantation model, it demonstrated that all of three printed CaP ceramics showed the ability of inducing ectopic bone formation, albeit weaker than the foamed ones. Moreover, macroporous structure played a critical role in determing the bioactivity of scaffolds, as the superior osteoinductivity was observed in Invert-fcc group with spherical, concave macropores similar to the foamed ceramics. Therefore, this study provides a promising strategy to fabricate osteoinductive CaP ceramics by using the DLP system and fully stimulating pore geometry of foamed ceramics or other biomimetic trabecular structure, in order to achieve desired clinical regeneration and repair effects.

## Supplementary data

Supplementary data are available at *REGBIO* online.

*Conflict of interest statement.* None declared.

## Supplementary Material

rbac005_Supplementary_Data
