# Supplementary material for: Fabrication and biological evaluation of 3D-printed calcium phosphate ceramic scaffolds with distinct macroporous geometries through digital light processing technology
Source: Regen Biomater. 2022 Feb 22;9:rbac005. doi: 10.1093/rb/rbac005 (PMC9160879; doi:10.1093/rb/rbac005)
Supplement: rbac005_Supplementary_Data [file rbac005_supplementary_data.doc]

Supplementary Table 1 (Table S1). Primer sequences for qRT-PCR assay

| Gene | 5' to 3' | Primers |
| --- | --- | --- |
| ALP | Sense | 5'-ATGGTAACGGGCCTGGCTACA-3' |
|  | Anti-Sense | 5'-AGTTCTGCTCATGGACGCCGT-3' |
| BMP-2 | Sense | 5'- AGTTACGAGCAAAGGCCTGA-3' |
|  | Anti-Sense | 5'-CGGTGTGACTCGTGCAGCCA-3' |
| BSP | Sense | 5'-CCAGCCAGGACTGCCGAAGG-3' |
|  | Anti-Sense | 5'-CGCTGCCTCCCTGGACTGGA-3' |
| OCN | Sense | 5'-GCTCAHCCTTCGTGTCCAAG-3 |
|  | Anti-Sense | 5'-GGGGGCTGGGGCTCCAAGT-3' |
| Runx 2 | Sense | 5'-AGATGGGACTGTGGTTACCG-3' |
|  | Anti-Sense | 5'-GGACCGTCCACTGTCACTTT-3' |
| OPN | Sense | 5'-CCTGGCAGGTGCAAAGCCCA-3' |
|  | Anti-Sense | 5'-GGGGGCTGGGGCTCCAAGT-3' |
| GAPDH | Sense | 5'-CCGTCGATCAGTTGGCGC-3' |
|  | Anti-Sense | 5'-TGGTGAAGACGCCAGTAGACTC-3' |
